# AntagomiR-451 inhibits oxygen glucose deprivation (OGD)-induced HUVEC necrosis via activating AMPK signaling

**DOI:** 10.1371/journal.pone.0175507

**Published:** 2017-04-26

**Authors:** Xi Yang, Xiao-Qing He, Guo-Dong Li, Yong-Qing Xu

**Affiliations:** 1 Department of Orthopedics, Kunming General Hospital, PLA, Kunming, China; 2 Brigade of Postgraduate Management, Third Military Medical University, Chongqing, China; Suzhou University, CHINA

## Abstract

Oxygen glucose deprivation (OGD) application in cultured human umbilical vein endothelial cells (HUVECs) mimics ischemic injuries. AntagomiR-451, the miroRNA-451 (“miR-451”) inhibitor, could activate pro-survival AMP-activated protein kinase (AMPK) signaling. In the current study, we showed that forced-expression of antagomiR-451 depleted miRNA-451 and significantly attenuated OGD-induced necrosis of HUVECs. Activation of AMPK was required for antagomiR-451-mediated pro-survival actions. AMPK inhibition, by AMPKα shRNA or dominant negative mutation, almost completely abolishedantagomiR-451-mediated HUVEC protection again OGD. Reversely, forced-activation of AMPK by exogenous expression of constructively-active AMPKα inhibited OGD-induced HUVEC necrosis. At the molecular level, antagomiR-451 expression in HUVECs inhibited OGD-induced programmed necrosis, the latter was evidenced by mitochondrial p53-cyclophilinD (Cyp-D) association, mitochondrial depolarization as well as reactive oxygen species (ROS) production and lactate dehydrogenase (LDH) breach. Together, we suggest that antagomiR-451 activates AMPK to inhibit OGD-induced programmed necrosis in HUVECs.

## Introduction

Ischemic vascular diseases cause substantial injuries to the vascular valve and vascular endothelial cells, which eventually leads to damages to surrounding tissues [1,2]. The underlying signaling mechanisms are still not fully understood [1,2]. Oxygen glucose deprivation (OGD) is applied to mimic ischemic damages *in vitro*. Treatment of vascular endothelial cells with serve and/or sustained OGD (>1 hour) will suppress mitochondrial respiratory chain. When coupled with re-oxygenation, superoxide and other reactive oxygen species (ROS) will be produced, causing oxidative stress and cell programmed necrosis (but not apoptosis) [3,4,5].

AMP-activated protein kinase (AMPK), the energy switcher, senses cellular AMP/ATP change and maintains balance of energy metabolism [6]. Recent studies have proposed the pro-survival function of AMPK under many stresses[7,8,9,10,11]. For instance, AMPK activation could act as an anti-oxidant protein via increasing intracellular NADPH(nicotinamide adenine dinucleotide phosphate) content[9,11]. AMPK phosphorylates and inhibits the major downstream target acetyl-CoA carboxylase (ACC), thus suppressing NADPH consumption[11]. It also promotes NADPH synthesis by fatty-acid oxidation [11]. Further, activated AMPK could also promote cell survival via initiating autophagy or suppressing mammalian target of rapamycin (mTOR)[7,8,9,10]. Recent studies have suggested that forced-activation of AMPK could also inhibit ischemic/OGD injuries[12,13].

microRNAs are 19–24 nucleotide single-stranded noncoding RNAs, which silence target mRNAs via partial complementarity in the untranslated regions (UTR)[14,15]. Very recent studies have suggested that microRNA-451 (“miR-451”) could be an AMPK inhibitor[16,17,18]. miR-451 silences calcium-binding protein 39 (CAB39, also known as MO25α), causing inhibition of AMPK kinase LKB1 (liver kinase B1), which eventually leads to AMPK in-activation [16,17,18]. Reversely, miR-451 depletion by expressing its inhibitor antagomiR-451 could lead to CAB39 accumulation and LKB1-AMPK activation[16,17,18]. In the current study, we show that antagomiR-451 expression inhibits OGD-induced human umbilical vein endothelial cell (HUVEC)necrosis via activating AMPK signaling.

## Materials and methods

### Chemicals and antibodies

The antibodies of this study were purchased from Cell Signaling Tech (Nanjing, China). Puromycin was obtained from Sigma-Aldrich (Shanghai, China). Cell culture reagents were provided by Gibco(Shanghai, China). Transfection reagents were obtained from Invitrogen (Shanghai, China).

### Cell culture

HUVECs were provided by the Hu’s group at Shanghai JiaoTong University School of Medicine [19]. HUVECs were cultured in routine medium 199with 15% FCS, and endothelial cell growth supplement (Sigma) plus epidermal growth factor (EGF 10 ng/mL)[19]. All experiments using human cells (HUVECs) were approved by the Institutional Review Board (IRB, No.BS200412) and Ethics Review Board (ERB, No.2013117) of Kunming General Hospital, PLA, and were conducted according to the principles expressed in the Declaration of Helsinki.

### OGD/re-oxygenation

OGD/re-oxygenation procedure was described in other studies [3,4,5,13,20]. Briefly, HUVECs were cultured under a pre-warmed glucose-free balanced salt solution (glucose deprivation) [3]. Cells was then bubbled with an anaerobic gas mix (95% N_2_, 5% CO_2_, oxygen deprivation). Cells were then maintained under OGD for 3 hours, and then re-oxygenated for indicated time.

### MTT assay of cell survival

After treatment, the viability of HUVECs was examined by the routine 3-[4,5-dimethylthylthiazol-2-yl]-2,5 diphenyltetrazolium bromide (MTT) assay [3,21]. Optic density (OD) value at 490 nM was recorded as the indicator of cell survival.

### LDH detection of cell necrosis

Cell necrosis was examined by routine lactate dehydrogenase (LDH) release assay using a commercial available two-step LDH kit (Takara, Tokyo, Japan). LDH release was calculated: LDH released in conditional medium/(LDH in conditional medium + LDH in cell lysates) × 100%.

### microRNA-451 and antagomiR-451 transfection

Similar as previously reported [18], transfection of pre-microRNA-451 (provided by Dr. Lu [17]), the antagomiR-451 (provided by Dr. Lu [17]) or the scrambled control antagomiR (“antagomiR-C”) was performed using the Lipofectamine 2000 reagents (Invitrogen). Transfection efficiency (>80%) was confirmed with the use of the Silencer FAM-labeled Negative Control (Ambion).

### Quantitative real-time polymerase chain reaction (qRT-PCR) assay

After treatment of cells, the total RNA was extracted via the Trizol reagents (Qiagen, Shanghai, China). The concentration of total RNA was quantified via a NanoDrop Spectrophotometer (NanoDrop Technologies). The TaqMan microRNA assay system was utilized to quantify miRNA-451 expression, using the Applied Biosystems 7500 Real-Time PCR System. ^ΔΔ^Ct method was employed to quantify mRNA expression. *GAPDH* was always tested as the internal control [22,23].miRNA-451 primers were provided by Dr. Lu [17].

### Western blotting assay

After treatment, the cell lysis buffer (Biyuntian, Wuxi, China) was applied to achieve the protein lysates. Twenty μg lysates per sample were separated by 8–10% SDS-PAGE gels, which were then transferred onto PVDF membranes (Shanghai, China). After blocking, the implied primary and secondary antibodies were then added. Afterwards, ECL reagents (Roche, Shanghai, China) were added to detect the interested bands. Tubulin was always tested as the loading control. ImageJ software was applied to quantify the total gray of each band.

### Mitochondrial immunoprecipitation (Mito-IP)

The detailed protocol was described previously[24,25,26,27]. Briefly, following treatment, the mitochondria of HUVECs were isolated via the “Mitochondria Isolation Kit” (Sigma) [3]. The pre-cleared mitochondrial lysates (0.5 mg per treatment) were incubated with anti-Cyp-D antibody or anti-p53antibody ([3,28]). The mitochondrial immune complexes were then captured with protein A/G-Sepharose (Sigma, Shanghai, China). Cyp-D-p53 association was then tested by Western blotting assay.

### Mitochondrial depolarization assay

Mitochondrial depolarization was tested using the fluorescence dye JC-10 (Invitrogen) [24,25,26,27,29]. Briefly, after indicated treatment, cells were stained with JC-10 (5.0 μg/mL), which was tested immediately on a spectrofluorometer to reflect intensity of mitochondrial depolarization.

### AMPKα shRNA

Two non-overlapping lentiviral AMPKα short hairpin RNAs (shRNAs), “shAMPKα (s1)” and “shAMPKα (s2)”, as well as the control scramble shRNA (“SCR shRNA”) were gifts from Dr. Lu [30,31,32]. The lentiviral shRNA was added directly to the cultured HUVECs, which were then subjected to puromycin (0.5 μg/mL, Sigma) selection for another 48 hours. AMPKα knockdown in HUVECs was verified by Western blotting assay.

### AMPKα mutation

The pSuper-puro construct with dominant negative AMPKα(T172A, DN-AMPKα, Flag-tagged), the constitutively-active AMPKα (T172D, ca-AMPKα, no tag), and the empty vector (pSuper-puro) were all from Dr. Lu[30,31,32]. The constructs were transfected to the HUVECs via Lipofectamine 2000 reagent (Invitrogen). Cells were then subjected to puromycin (0.5 μg/mL) selection for additional 48 hours. Expression of the mutant AMPKα was confirmed by Western blotting assay.

### ROS assay

The intracellular ROS content was tested by the dichlorofluorescin (DCF) oxidation assay, and the detailed protocol was described previously [33]. Following the applied treatment, 10 μM DCFH-DA (Invitrogen) was added. Cells were then washed, trypsinized and resuspended in PBS. DCF fluorescence was then examined. The excitation wavelength was set at 488 nm. The DCF fluorescent OD value of treatment group was always normalized to that of untreated control group.

### Lipid peroxidation assay

Thiobarbituric acid reactive substances (TBAR) level was tested to reflect lipid peroxidation, the detailed protocol was described previously [34]. Briefly, after treatment, intracellular lysates (20 μg per treatment) were mixed with acetic acid (20%) and aqueous solution of thiobarbituric acid (0.78%). After heating, the mixtures were centrifuged, and then the red pigment in the supernatant was estimated by a microplate reader. The lipid peroxide level was expressed as nM of malondialdehyde per mg protein. The values of treatment group were always normalized to that of control group.

### Statistics

The data were presented as mean ± standard deviation (SD). Statistical differences were analyzed by one-way ANOVA with post hoc Bonferroni test (SPSS version 18.0). Values of *p*< 0.05 were considered statistically significant.

## Results

### AntagomiR-451 expression attenuates OGD-induced necrosis of HUVECs

**Fig 1:**
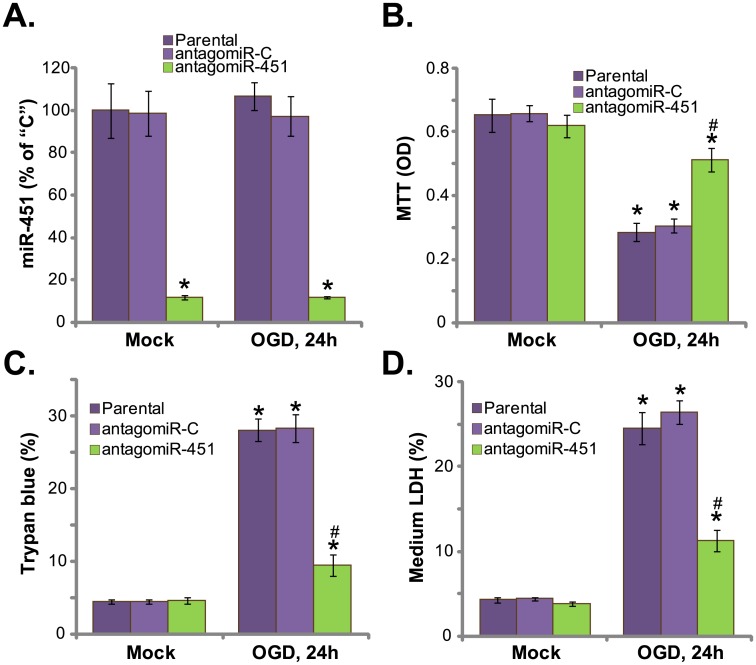
AntagomiR-451 expression attenuates OGD-induced necrosis of HUVECs. Parental HUVECs (“Parental”) as well as HUVECs expressing antagomiR-451 or antagomiR-C were maintained under OGD for 3 hours, followed by 24 hours of re-oxygenation;miR-451 expression was tested by qRT-PCR assay (A); Cell survival was tested by MTT assay (B); Cell death was examined by Trypan blue staining assay (C); LDH release in conditional medium was also measured as indicator of cell necrosis (D). “Mock” stands for normal culture condition (Same for all figures). “OGD” stands for OGD/re-oxygenation (Same for all figures). Bars indicate mean ± standard deviation (SD, n = 5). *p<0.05 *vs*. “Mock”. ^**#**^*p*<0.05 *vs*. “OGD” of “antagomiR-C” cells. Each experiment was repeated five times and similar results were obtained.

First, HUVECs were cultured with OGD for 3 hours(see Methods), followed by re-oxygenation for another 24 hours. qRT-PCR assay results in Fig 1A demonstrated that miR-451 level in HUVECs was unchanged before and after OGD treatment. Expression of antagomiR-451[17] caused dramatic decrease of miR-451 in HUVECs (Fig 1A). Significantly, OGD-induced death of HUVECs, evidenced by MTT viability OD reduction (Fig 1B) and Trypan blue positive cell increase (Fig 1C), was largely attenuated in antagomiR-451-expressing cells. Therefore, miR-451 depletion by antagomiR-451 protected HUVECs from OGD (Fig 1B and 1C).Results in Fig 1D showed that OGD treatment in HUVECs induced significant LDH release to the medium, which is a characteristic marker of cell necrosis. Such effect by OGD was again inhibited by antagomiR-451 (Fig 1D). AntagomiR-C, the control antagomiR, had no significant effect on miR-451expression (Fig 1A) and OGD-induced HUVEC death (Fig 1B–1D). Using multiple apoptosis assays, including Annexin V FACS assay and TUNEL staining assay, we failed to detect significant apoptosis in OGD-treated HUVECs, which was in line with other studies[3,4,5].Together, these results demonstrate that antagomiR-451 expression depletes miR-451 and attenuates OGD-induced HUVEC necrosis.

### AMPKα knockdown abolishes antagomiR-451-medaitd HUVEC protection again OGD

**Fig 2:**
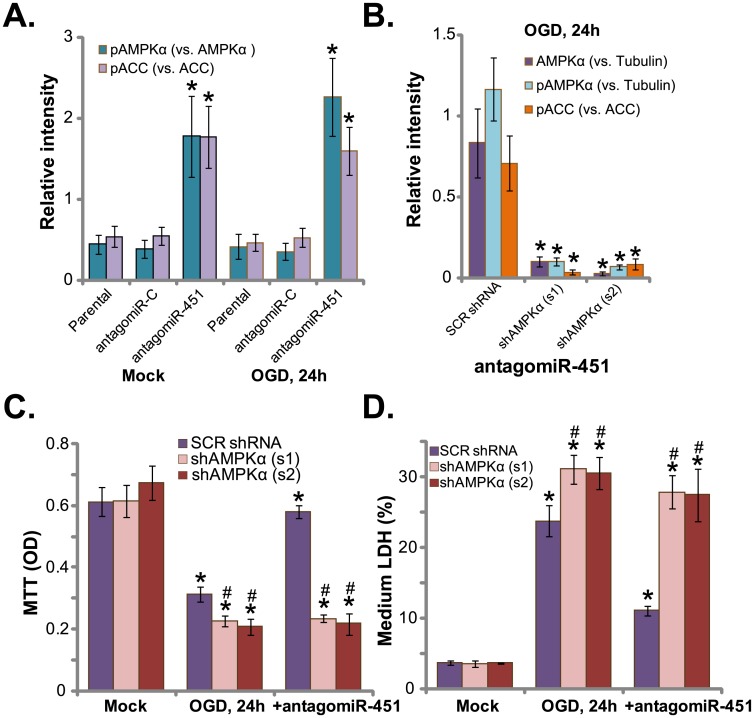
AMPKα knockdown abolishes antagomiR-451-medaitd HUVEC protection again OGD. Parental HUVECs (“Parental”) as well as HUVECs expressing antagomiR-451 or antagomiR-C were maintained under OGD for 3 hours, followed by 24 hours of re-oxygenation, p-/total AMPKα and ACC were shown, blot data were quantified (A).HUVECs, with/outantagomiR-451, were infected with lentiviral scramble control shRNA (“SCR shRNA”) or AMPKα shRNA (“s1/s2”); Cells were then subjected to the OGD for 3 hours, followed by 24 hours of re-oxygenation; Expressions of p-/total AMPKα and ACC were shown, blot data were quantified (B); Cell survival and necrosis were tested by MTT assay (C) and LDH release assay (D), respectively. Bars indicate mean ± standard deviation (SD, n = 5).*p<0.05 *vs*.“Parental” cells (A).*p<0.05 *vs*. group “SCR shRNA” (B).*p<0.05 *vs*. group “Mock” (C and D). ^**#**^*p*<0.05 *vs*.“SCR shRNA” group(C and D).Each experiment was repeated four times and similar results were obtained.

miR-451 could function as an AMPK inhibitor[17]. On the other hand, antagomiR-451 could then activate AMPK signaling via depletingmiR-451[17]. We thus tested AMPK signaling in antagomiR-451-expressing cells. Quantified blot results (n = 5) in Fig 2A showed that AMPK activation, tested by p-AMPKα (Thr-172) and p-ACC (Ser-79, a major downstream effector protein of AMPK), was indifferent before and after OGD treatment in HUVECs. Yet, HUVECs with antagomiR-451showed significant increased AMPK activation (p-AMPKα/ACC increase, Fig 2A). To study the link between HUVEC protection and AMPK activation. shRNA strategy was utilized to knockdown AMPKα. Two lentiviral AMPKα shRNAs, which targeting non-overlapping sequences of AMPKα, were applied. Results demonstrated that the two shAMPKα(s1/s2)[30,32,35], induced dramatic AMPKα downregulation (quantified blot results, Fig 2B, n = 5).Consequently, antagomiR-451-induced AMPK activation, or p-AMPKα/p-ACC, was almost completely blocked(quantified blot results, Fig 2B, n = 5). AntagomiR-451-medaited HUVEC protection against OGD was almost nullified in AMPKα-silenced cells (Fig 2C and 2D).Thus, antagomiR-451 was in-effective against OGD when AMPKα was silenced(Fig 2C and 2D).These results suggest that activation of AMPK is required for antagomiR-451-medaitd anti-OGD activity. Notably, HUVECs with AMPKα shRNA were more vulnerable to OGD(Fig 2C and 2D). These results imply that basal AMPK activation should also be pro-survival against OGD.

### AMPKα dominant negative mutation abolishesantagomiR-451-medaitd HUVEC protection again OGD

**Fig 3:**
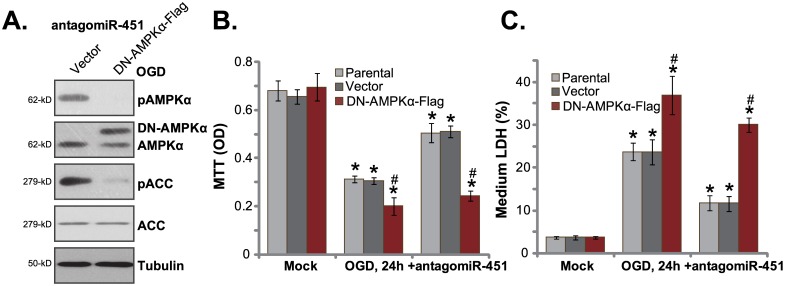
AMPKα dominant negative mutation abolishes antagomiR-451-medaitd HUVEC protection again OGD. HUVECsexpressingantagomiR-451 were infected with lentiviral dominant negative AMPKα (T172A, “DN-AMPKα”) or empty vector (“Vector”, pSuper-puro-Flag), expressions of listed proteins were shown (A); Above cells or parental cells were also subjected to the OGD, cell survival and necrosis were tested by MTT assay (B) and LDH release assay (C), respectively. Bars indicate mean ± standard deviation (SD, n = 5).*p<0.05 *vs*. group “Mock”. ^**#**^*p*<0.05 *vs*.“Vector” group. Each experiment was repeated three times and similar results were obtained.

Based on the above results, we propose that AMPK in-activation should also block antagomiR-451-medaitd HUVEC protection. Thus, a dominant negative AMPKα (T172A, “DN-AMPKα”)[30,31,32] was introduced to HUVECs. Western blotting assay results in Fig 3A confirmed expression of the exogenous DN-AMPKα (Flag-tagged) in antagomiR-451-expressing cells. Notably, introduction of DN-AMPKα in HUVECs almost completely blocked antagomiR-451-induced AMPK activation or p-AMPKα/p-ACC (Fig 3A). As a result, antagomiR-451-mediated HUVEC cytoprotection against OGD was also nullified (Fig 3B and 3C).HUVECs with antagomiR-451 were unable to resistant to OGD when AMPKα was in-active (Fig 3B and 3C). These results again suggest that activation of AMPK is indeed indispensable for antagomiR-451-mediated cytoprotection in HUVECs. It should be noted that OGD-induced death of HUVECs was again aggravated with DN-AMPKα expression(Fig 3B and 3C), once again confirming that basal AMPK activation is pro-survival in HUVECs.

### Expression of miR-451 potentiates OGD damages to HUVECs

**Fig 4:**
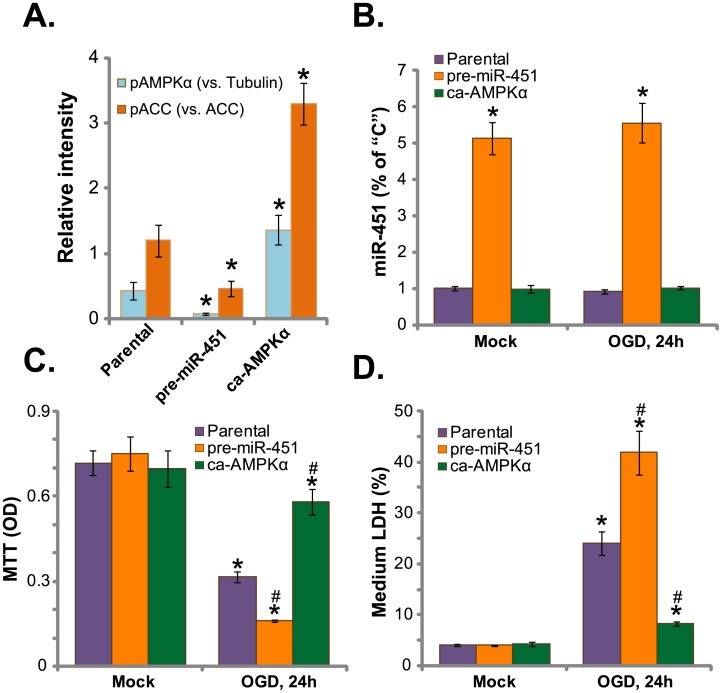
Expression of pre-miR-451 potentiates OGD damages to HUVECs. HUVECs with pre-miR-451, the constitutively-active AMPKα (T172D, “ca-AMPKα”) or the parental cells, were treated with/out OGD, expressions of listed proteins were tested, and blot data were quantified (A); miR-451 expression was tested by qRT-PCR assay (B); Cell survival and necrosis were tested by MTT assay (C) and LDH release assay (D), respectively.Bars indicate mean ± standard deviation (SD, n = 5).*p<0.05 *vs*. group “Parental” cells (A).*p<0.05 *vs*. group “Mock” (B-D). ^**#**^*p*<0.05 *vs*.“Parental” cells (B-D). Each experiment was repeated three times and similar results were obtained.

Next, pre-miR-451[17] was transfected to the HUVECs (Fig 4A). The miR-451 expression level was significantly increased in HUVECs with pre-miR-451, regardless of OGD treatment (Fig 4B). Notably, pre-miR-451 expression further inhibited basal AMPK activation (p-AMPKα/p-ACC) in HUVECs (quantified blot results, Fig 4A, n = 5). Consequently, OGD-induced HUVEC viability reduction (Fig 4C) and necrosis (Fig 4D), were largely potentiated. Thus, pre-miR-451 expression inhibited AMPK activation and potentiated OGD-induced death of HUVECs. On the other hand, the constitutively-active AMPKα (T172D, “ca-AMPKα”) [30,32,35] was introduced to HUVECs. Expectably, AMPK activation, or p-AMPKα/p-ACC, in HUVECs was augmented after ca-AMPKα expression(quantified blot results, Fig 4A, n = 5). Similar to the actions of antagomiR-451, ca-AMPKα significantly attenuated OGD-induced HUVEC viability reduction (Fig 4C) and necrosis (Fig 4D). Notably, ca-AMPKα didn’t affect miR-451 level in HUVECs (Fig 4B). Thus, ca-AMPKα activated AMPK and inhibited OGD-induced death of HUVECs.

### AntagomiR-451 attenuates OGD-induced programmed necrosis in HUVECs

**Fig 5:**
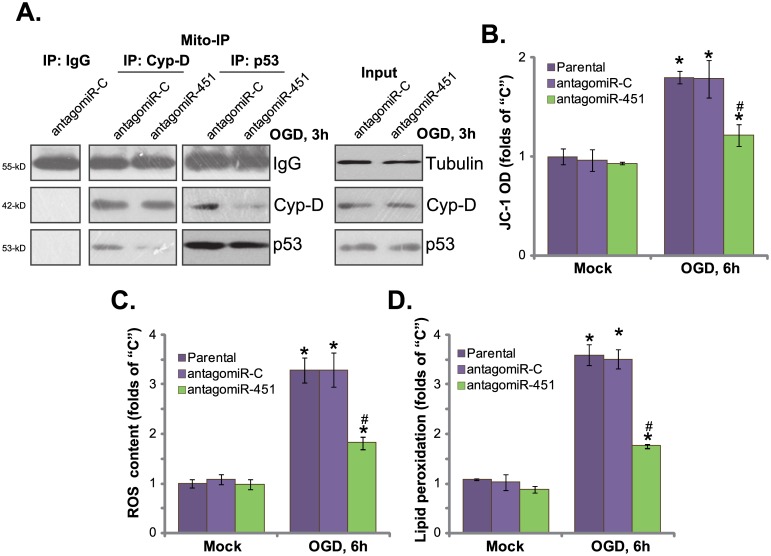
AntagomiR-451 attenuates OGD-induced programmed necrosis in HUVECs. HUVECs expressing antagomiR-451 or antagomiR-C were maintained under OGD for 3 hours, followed by re-oxygenation for indicated time, mitochondrial fraction was isolated, and the association between Cyp-D and p53 was tested by the co-immunoprecipitation assay (“Mito-IP”) assay (A, left panel). “Input” tested Cyp-D and p53 expression (A, right panel);Mitochondrial depolarization was tested by JC-10 assay (B); Relative ROS content (C) and lipid peroxidation level (D) were also tested. Bars indicate mean ± standard deviation (SD, n = 5).*p<0.05 *vs*. group “Mock”. ^**#**^*p*<0.05 *vs*. “OGD” of “antagomiR-C” cells. Each experiment was repeated three times and similar results were obtained.

Recent studies have implied that OGD mainly induces cell programmed necrosis, but not apoptosis[4,13].OGD induces p53 translocation to mitochondria, where it associates with local protein cyclophilinD (Cyp-D) [3,4,5,28]. The complexation causes mitochondrial depolarization, ROS production and cell necrosis (but not apoptosis) [3,4,5,28]. The activation of programmed necrosis was also noticed in OGD-treated HUVECs, evidenced by mitochondrial p53-Cyp-D association (Fig 5A) and mitochondrial depolarization (JC-10 OD increase[4,36,37], Fig 5B). Further, ROS level(Fig 5C) and lipid peroxidation (Fig 5D) were both increased following OGD in HUVECs, indicating oxidative stress. Such effects by OGD were largely inhibited after expressing antagomiR-451 (Fig 5A–5D). AntagomiR-451 inhibited OGD-induced mitochondrialp53-Cyp-D association (Fig 5A),mitochondrial depolarization (Fig 5B), and oxidative stress (Fig 5C and 5D) in HUVECs. Therefore, antagomiR-451 attenuated OGD-induced programmed prognosis in HUVECs.

## Discussion

The potential activity of miR-451 in various cancer cells has been extensively studied [38,39,40,41]. It has been shown that miR-451 level was decreased in human cancer, which serves as a potential tumor suppresser [38,39,40,41]. Over-expression of miR-451 could inhibit cancer cell survival and proliferation via silencing different oncogenic proteins [38,39,40,41,42]. In the current study, we showed that miR-451 expression potentiated OGD damages to HUVECs. On the other hand, antagomiR-451 expression inhibited OGD-induced HUVEC necrosis by activating AMPK signaling.

We showed that activation of AMPK was required for antagomiR-451-mediated HUVEC cytoprotection. AMPK inhibition, by AMPKα shRNA or dominant negative mutation, almost abolishedantagomiR-451-medaitd HUVEC protection again OGD. On the other hand, forced activation of AMPK by exogenous expression of ca-AMPKα inhibited OGD-induced death of HUVECs. Remarkably, pre-miR-451 expression led to further AMPK inhibition, thus potentiating death of HUVECs by OGD. Thus, AMPK is indeed pro-survival again OGD in HUVECs. Expression antagomiR-451 activated AMPK to inhibit ODG-induced necrosis of HUVECs.

The pivotal role of p53 in mediating cell apoptosis has been well-established. Very recent studies have also implied the requirement of p53 in promoting non-apoptotic cell death, known as programmed necrosis [28]. Under OGD, activated p53 is shown to trigger mitochondrial permeability transition pore (mPTP) opening via physically interact with Cyp-D in mitochondria, thus inducing mitochondrial depolarization, ROS production and cell necrosis (but not apoptosis) [3,28]. Inhibition or silence this complex was shown to attenuate or even reverse OGD-induced programmed necrosis [3,4,5,28]. In line with these studies, OGD also provoked mitochondrial necrosis pathway in HUVECs, evidenced by mitochondrial p53-Cyp-D association, mitochondrial depolarization and profound ROS production. Our results here demonstrated thatantagomiR-451expression in HUVECs largely inhibited OGD-induced programmed necrosis, which might explain the superior pro-survival activity of antagomiR-451 in HUVECs.

## Conclusion

AntagomiR-451 inhibits OGD-induced necrosis of HUVECs possibly via activating AMPK signaling.
